# Medical Record Review to Differentiate between Idiopathic Parkinson's Disease and Parkinsonism: A Danish Record Linkage Study with 10 Years of Follow-Up

**DOI:** 10.1155/2015/781479

**Published:** 2015-12-03

**Authors:** Lene Wermuth, Xin Cui, Naomi Greene, Eva Schernhammer, Beate Ritz

**Affiliations:** ^1^Department of Neurology, Odense University Hospital, Odense, Denmark; ^2^Department of Epidemiology, University of California, Los Angeles, School of Public Health, Los Angeles, CA, USA; ^3^Channing Division of Network Medicine, Department of Medicine, Harvard Medical School, Boston, MA, USA; ^4^Department of Epidemiology, Harvard School of Public Health, Boston, MA, USA; ^5^Department of Epidemiology, Center for Public Health, Medical University of Vienna, Vienna, Austria

## Abstract

*Background*. The electronic medical records provide new and unprecedented opportunities for large population-based and clinical studies if valid and reliable diagnoses can be obtained, to determine what information is needed to distinguish idiopathic PD from Parkinsonism in electronic medical records.* Methods*. Chart review of complete medical records of 2,446 patients with a hospital discharge diagnosis of PD, who, between 1996 and 2009, were registered in the Danish National Hospital Register as idiopathic PD. All patients were examined in neurology departments. Clinical features were abstracted from charts to determine Parkinsonian phenotypes and disease course, using predefined criteria for idiopathic PD.* Results*. Chart review verified that 2,068 (84.5%) patients met criteria for idiopathic PD. The most distinguishing features of idiopathic PD patients were asymmetric onset, and fewer atypical features at onset or follow-up compared to Parkinsonism, and the area under the curve (AUC) for these items alone is moderate (0.74–0.77) and the highest AUC (0.91) was achieved when using all clinical features recorded in addition to PD medication use and a follow-up of 5 years or more.* Conclusion*. To reduce disease misclassification, information extracted from medical record review with at least 5 years of follow-up after first diagnosis was key to improve diagnostic accuracy.

## 1. Introduction

Etiologic studies of idiopathic Parkinson's disease (IPD) and the identification of predictors of progression or severity necessitate finding and following PD patients preferably in a population-based manner. The growth of electronic disease registration and medical record systems may aid such studies. However, establishing valid and reliable diagnoses is a challenge since IPD shares symptoms with a number of other diseases commonly referred to as Parkinsonism such as atypical and secondary Parkinsonism [1]. Despite differences in etiology and course of treatment these phenotypes are not easily distinguishable from each other, especially early in disease. To aid large scale, cost efficient, and timely medical and pharmaceutical record-based studies of PD, we describe here how—according to medical records from neurologic departments in Denmark—clinical features develop over time and may help in assessing the accuracy of an IPD diagnosis [2, 3]. While the most definite IPD diagnosis is made at autopsy, few patients are assessed postmortem and almost all studies have to rely on clinical diagnoses [1]. Clinicopathological studies suggested an error rate of 10–25% in diagnosing IPD with lower misdiagnosis among movement disorder neurologists [4].

A classic study named the absence of atypical features, asymmetric onset, and absence of listing of extraneous causes for Parkinsonian syndromes as best predictors of pathologically proven IPD [5]. Disease duration and responsiveness to PD medications have also been considered: using a neuropathologic gold standard diagnostic accuracy of “responsiveness to medication” was only 53% in early IPD and increased to more than 85% after 5 years of disease duration [6]. Two epidemiological studies in the United Kingdom reported on the accuracy of IPD diagnoses in community-based studies. In greater London, researchers screened medical records from 15 general practices and for 202 PD patients after review of the diagnosis was rejected for 15% [7]. Among 128 patients identified from general practitioners records in Scotland, 11% received a revised diagnosis of essential tremor and 8% of vascular Parkinsonism after clinical examination [2].

Here we use data from the Danish health system to evaluate diagnostic accuracy of electronic records with a primary ICD for IPD. Since 1 January 1977, the Danish National Hospital Register (NHR) electronically records inpatient-related services and diagnoses from all hospitals and for all citizens using a unique Danish citizen identification number. Outpatient contacts have been added since 1 January 1994 [8]. We screened the NHR for PD ICD codes as primary diagnosis and retrieved medical records from neurology centers to assess information on major symptoms and signs of PD present at the time of diagnosis and at subsequent hospital visits. In a pilot study, we previously reviewed records of 1,040 PD patients reported to the NHR and found that only 82% suffered from IPD [9]. We now present data for 2,446 patients from 10 major neurologic centers in Denmark with a primary diagnosis of IPD from the NHR in 1996–2009 for whom we collected baseline and follow-up information on clinical PD features through a complete medical record review. We evaluate how clinical features necessary for deriving an IPD diagnosis are presented at onset and over time and, for the first time, also describe treatment regimens and compare comorbidities and vital status changes of patients and compare those to population controls [10].

## 2. Material and Methods

We identified patients with a primary diagnosis of IPD (ICD-8 code 342, ICD-10 code G20) at hospital discharge age 35 or older from the Danish NHR between 1996 and 2009. At diagnosis, patients had to be 70 years of age or less before 2002 and 80 years of age or less in 2002–2009 to ensure that most eligible patients survived to planned interviews in 2007–2009 (for further details see [11]). To increase diagnostic validity, only patients treated at any time in 10 (*N* = 3,508) out of 15 major neurologic centers (*N* = 4,975) were eligible. We did not attempt to retrieve medical charts for patients who died before contact for interview (*n* = 362), for whom a “research protection” prohibited contact (*n* = 156), who lacked contact information (*n* = 110), or were too ill to participate (*n* = 115) or unable to speak English or Danish (*n* = 3). From among the 2,762 patients left, we excluded 179 for whom an initial brief screening of charts excluded IPD and 137 who refused interviews or lacked medical records. In total 2,446 medical charts were reviewed and signs and symptoms necessary to establish an IPD diagnosis according to United Kingdom Brain Bank and Gelb criteria abstracted [1, 12]; note that the first 1000 charts were retrieved and reviewed without consideration of patient refusal of contact or death prior to interview [9]. The final diagnosis of treating neurologic specialists was recorded, but we made a diagnosis based on all of the medical record information available and required the presence of a minimum of two of the following symptoms: resting tremor, bradykinesia, rigidity, and asymmetrical onset. We also abstracted notes from private practitioners who often treat patients before hospital/clinic admissions and reviewed notes about treatment courses during inpatient stay or outpatient clinic visits. We recorded age at first self-reported symptom, patients' response to treatment with levodopa, signs of or test results for dementia, as well as early falls, severe symptomatic dysautonomia, and sudden symptom onset, supranuclear gaze palsy, hallucinations unrelated to medication, freezing phenomena, Babinski's sign, and symptoms for other brain/nervous system diseases, and records for computed tomography scans, DaTSCANs, or magnetic resonance scans. Almost all patient medical charts included a computed tomography scan and/or a magnetic resonance scan and 30% of records also contained a DaTSCAN and this information was employed in the validation of the diagnosis. We selected controls matched to cases on birth year and gender.

To comparing cases according to clinical features, we used chi-square tests and calculated for each the sensitivity, specificity, and positive and negative predicted values for a diagnosis of IPD. To determine the best combination of IPD predictors, we plotted ROC curves and determined the area under the curve (AUC) for four different prediction models. Specifically, model 1 (full model) included all typical clinical and atypical features, asymmetrical onset, and PD medication use listed in Table 4. The reduced model 2 includes only atypical features and one major cardinal symptom: asymmetric onset. Model 3 (maximum atypical features model) was restricted to atypical features only; and model 4 (minimum atypical features model) dropped severe autonomic dysfunction and supranuclear gaze palsy from atypical features.

## 3. Results

Out of 2,446 patients with a primary IPD diagnosis, we determined that 378 (15.5%) did not have IPD (compared with 694 (25%) out of the initial 2,762 prior to initial record screening), leaving 2,068 patients with IPD according to our criteria and information provided in charts. Among the 378 cases who did not have IPD with a neurologic hospital register based ICD code for PD there were 118 patients with atypical PD (53 with LBD, 44 with MSA, and 21 with PSP or CBD (tauopathies)), 125 with secondary or other types of Parkinsonism, and 35 with essential tremor (ET) and 100 had an incomplete chart preventing us from establishing a diagnosis. Out of 2,446 patients, 733 (30%) had received a DaTSCAN that identified 131 as non-IPD even though they were recorded as IPD in the NHR. Atypical and secondary Parkinsonism patients while born earlier had later disease onset; that is, they were older at time of diagnosis than IPD patients and more likely to die between study enrolment and end of follow-up (2007–2013) (Table 1). The three cardinal PD signs (tremor, rigidity, and bradykinesia) and PD medication use frequencies at diagnosis did not differ between phenotypes (50–60% prevalence). However, asymmetric onset was recorded for a significantly higher proportion of IPD patients (88.3% versus 64.3% non-IPD) (Table 2). During follow-up, 85–96% of all IPD patients developed the three cardinal signs and 99% took PD medications, while 63–74% of non-IPD patients developed these signs and only 80% received PD medications.

**Figure 1:**
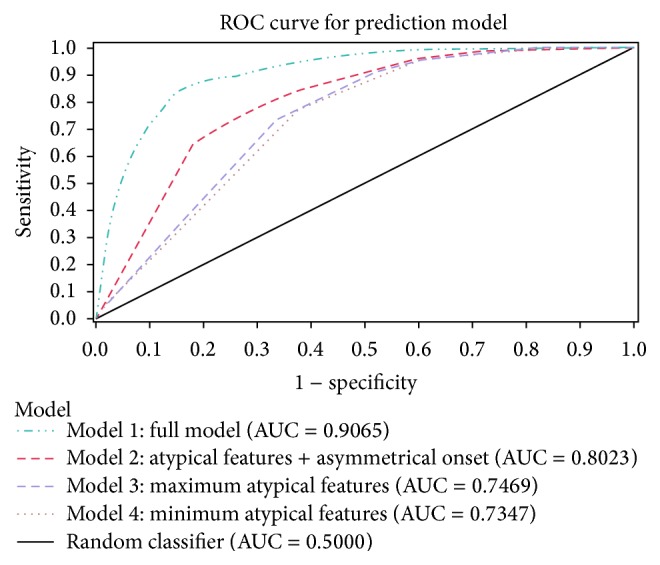
ROC curves for diagnosing iPD based on symptoms and medications (ever listed in medical records).

More non-IPD (13.2%) than IPD (7.4%) patients took antidepressant medication prior to or at diagnosis; there also were large differences in comorbidities prior to diagnosis (Table 3). Compared to population controls, a higher percentage of IPD patients suffered from heart disease, CVD, and dementia but less had a COPD diagnosis. Clinical features and PD medication use within the first year of diagnosis, had high sensitivity (66% to 89%) but low specificity even when combining features (18% to 57%) (Table 4). Using all chart information throughout follow-up (on average 6.6 years after PD diagnosis) the AUC for distinguishing IPD from non-IPD was high (0.91) (Figure 1) but reduced to 0.82 when relying on these features (from Table 4) observed within the first year of diagnosis only and the AUC dropped to 0.68 or 0.69 in the reduced model 3 or 4.

## 4. Discussion

Parkinson's registries similar in coverage or accuracy to cancer registries do not exist, but electronic medical records hold promise for identifying IPD patients and would be of great value for large scale population-based and clinical studies. Few studies have described symptoms and phenotypic features of PD at onset and during progression in a population-based sample [13, 14] and none is as large a study as ours. Many clinical trials, etiologic studies, or surveillance studies select patients early in disease to capture incident patients and limit recall bias but are unable to follow-up and reassess diagnoses over extended periods of time. Identifying incident IPD cases shortly after diagnosis even from neurologic centers invites misdiagnosis. Our data suggest that at least 15–25% of IPD diagnoses based on ICD codes early in disease are inaccurate, likely a conservative estimate, since we only relied on neurologic speciality clinic patients expected to have higher clinical accuracy [4]. This is supported by data from a highly specialized tertiary care clinic in the US, which reported that 8.1% of 800 patients initially diagnosed with IPD were reclassified after 7.6 years of follow-up [15]. A smaller study using neuropathologic findings confirmed IPD in only 53% of cases within <5 years of disease compared with >85% diagnostic accuracy after longer disease duration [6].

General clinical wisdom holds that patients with IPD more often experience tremor and asymmetric onset, while atypical PD patients more often suffer from bradykinesia, early falls, and severe dysautonomia, while secondary Parkinsonism might have sudden symptoms onset [1]. However, our chart review showed that at diagnosis (or first visit to a neurologic clinic reporting to the NHR) there was no difference in the frequency of three cardinal symptoms, postural instability was present in less than a quarter, and atypical features were present in only 10-11% of all non-IPD cases, confirming that based on clinical features, even in neurologic centers, it is difficult to make an accurate diagnosis of IPD early in disease.

Moreover, many different forms of non-IPD Parkinsonism exist. For example, multiple systemic atrophy (MSA) is known for dysautonomia and cerebellar and corticospinal deficits [16, 17]. Patients with progressive supranuclear palsy (PSP) show often symmetric onset with early falls, and vertical supranuclear gaze palsy [18]. In dementia with Lewy Body (LBD) cognitive impairment, often combining fluctuating cognition and recurrent visual hallucinations, precedes motor symptoms [16]. Corticobasal degeneration (CBD) is difficult to clinically differentiate from other types of atypical Parkinsonism [19]. Secondary Parkinsonism includes medication-induced or vascular syndromes. Vascular Parkinsonism may start abruptly with predominant lower body involvement and postural instability [16] but might show in neuroimaging. A recent study reported that out of 16 cases for whom PD could not be confirmed neuropathologically one had MSA and 7 PSP, 5 various neurodegenerative findings, and 3 no findings to explain the Parkinsonism [6]. In sum, while clinical features may distinguish IPD and atypical and secondary PD in the long run, our data corroborate the notion that differential diagnoses are not likely to be accurate in the first 5 years after onset. More than half of our patients had a PD ICD diagnosis in the NHR system for more than 10 years at the time of chart review. When relying solely on clinical diagnosis, it might be necessary to abandon the emphasis on an early diagnosis to recruit for incident studies or to conduct additional follow-up and revise ICD classifications since additional features vastly improve diagnostic accuracy. Of interest, patients for whom our record review resulted in reclassifications to atypical or secondary PD tended to be older and had more comorbid diseases, and 74–100% of them died during follow-up by 2013.

Some countries have prescription drug databases available that offer the opportunity to identify PD patients for studies. In our study, PD medications were consumed by about half of all IPD and non-IPD patients at time of diagnosis and almost 100% of IPD patients received PD medications eventually, compared with 80% of non-IPD patients. Hence, the best time to identify PD patients based on medications alone might also be more than 5 years after diagnosis, at which time 92% of IPD and 76% of non-IPD patients have received some treatment and it can be determined whether treatment was discontinued—a possible indication of no benefit as seen for atypical Parkinsonism.

While levodopa is still the most commonly prescribed medication, in Denmark many start treatment with a dopa-agonist and/or MAO-B inhibitor first, likely due to recommendations for younger (<60 years) or less severe PD patients to avoid levodopa-related motor complication [20]. Older patients with more atypical symptoms early on may be started on levodopa supplemented with other medications later. Lastly, patients with atypical PD may be responding less to and receive smaller doses of levodopa [21]. Hence, such treatment patterns could potentially aid in improving diagnostic accuracy for electronic record only based studies.

A major and to date elusive goal of PD research is to develop neuroprotective or disease-modifying therapies that can stop or slow disease progression. Enrolling patients early into trials leads to different phenotypes being mixed together. Nondopaminergic symptoms such as postural instability, autonomic dysfunction, and dementia at diagnosis have been considered main predictors of faster progression and disability in the first five years of disease [14], but our data demonstrate that these symptoms are observed in less than 50% of non-IPD patients and less than 20% of IPD cases within 5 years of diagnosis. Improving diagnostic accuracy based on medical record data is essential for future record linkage studies to be useful in identifying new etiologic factors and treatment modalities and for targeting disease subtypes and progression.

In summary, our record review of patients with an ICD code for IPD as a primary diagnosis in the Danish NHR confirmed that only 75–85% of the patients originally selected for study suffered from IPD. Thus, our data suggest that electronic data sources need to be developed that provide information not only on ICD codes but also on cardinal and atypical symptoms, symmetry of onset, comorbidities, and treatment modalities for all PD patients. To be most informative in terms of predictive validity for IPD, these data need to be used in combination and patients need to be followed up over at least 5 years into disease to improve diagnostic accuracy in studies that rely solely on record linkage.

## Supplementary Material

Fig. 1. shows the ROC curves for diagnosing IPD after 1 year based on symptoms and PD medications.
Table 1. shows the diagnostic value of clinical features in IPD cases and non-IPD cases based on symptoms and medications.
Table 2. shows the medical treatment registered in medical records at any time.

## Figures and Tables

**Table 1:** Demographic characteristics of participants in the Danish PASIDA study (for PD cases according to final type of Parkinsonism determined by medical record review).

	IPD		LBD		MSA		PSP & CBD		Secondary & other types of Parkinsonism		Essential tremor		Enrolled controls	
	*N*	%	*N*	%	*N*	%	*N*	%	*N*	%	*N*	%	*N*	%
Total	2068	100.0	53	100.0	44	100.0	21	100.0	125	100.0	35	100.0	1887	100.0
Gender														
Male	1228	59.4	36	67.9	27	61.4	11	52.4	62	49.6	23	65.7	1121	59.4
Female	840	40.6	17	32.1	17	38.6	10	47.6	63	50.4	12	34.3	766	40.6
Year of birth														
1920–1929	199	9.6	7	13.2	6	13.6	1	4.8	31	24.8	7	20.0	162	8.6
1930–1939	872	42.2	33	62.3	17	38.6	13	61.9	54	43.2	17	48.6	785	41.6
1940–1949	712	34.4	13	24.5	17	38.6	5	23.8	27	21.6	10	28.6	655	34.7
1950–1959	227	11.0	0	0.0	4	9.1	2	9.5	7	5.6	1	2.9	224	11.9
From 1960 on	58	2.8	0	0.0	0	0.0	0	0.0	6	4.8	0	0.0	61	3.2
PD onset in calendar year^a^														
1986–1994	303	14.7	0	0.0	2	4.5	0	0.0	8	6.4	2	5.7	NA	NA
1995–2004	1148	55.5	25	47.2	26	59.1	8	38.1	76	60.8	21	60.0	NA	NA
From 2005 on	617	29.8	28	52.8	16	36.4	13	61.9	41	32.8	12	34.3	NA	NA
Age at PD diagnosis^b^														
36–45	86	4.2	0	0.0	1	2.3	0	0.0	10	8.0	0	0.0	81	4.3
45–54	304	14.7	2	3.8	6	13.6	3	14.3	4	3.2	1	2.9	279	14.8
55–64	757	36.6	6	11.3	13	29.5	5	23.8	34	27.2	9	25.7	680	36.0
65–74	707	34.2	34	64.2	12	27.3	11	52.4	47	37.6	17	48.6	650	34.4
75–80	214	10.3	11	20.8	12	27.3	2	9.5	30	24.0	8	22.9	197	10.4
Vital status														
Dead between end of 2007 and 2010	213	10.3	10	18.9	16	36.4	8	38.1	24	19.2	2	5.7	31	1.6
Dead between end of 2007 and 2013	458	22.1	29	54.7	27	61.4	13	61.9	49	39.2	7		111	5.9

^a^PD onset was defined as year of first symptom onset (resting tremor, bradykinesia, rigidity, asymmetry, postural reflex impairment, or unspecific/self-reported symptom) as reported in medical records; when dates were missing for all symptoms, PD onset was defined as year of first ever PD diagnosis recorded in the NHR.

^b^Age at PD diagnosis was based on the year of first ever PD diagnosis in the NHR; for controls, it was the age at PD diagnosis of their matched case.

**Table 2:** PD related symptoms reported in medical records among idiopathic and non-IPD cases at onset and during follow-up.

Symptom	Any time		Before/at time of diagnosis		≤1 year after diagnosis		>1–5 years after diagnosis		>5–10 years after diagnosis		≥10 years after diagnosis	
	*N*	%	*N*	%	*N*	%	*N*	%	*N*	%	*N*	%
IPD (*N* = 2,068)^a,c,d^												
Tremor	1750	84.6	1250	60.4	145	7.0	217	10.5	78	3.8	58	2.8
Rigidity	1977	95.6	1121	54.2	231	11.2	359	17.4	155	7.5	105	5.1
Bradykinesia	1932	93.4	1090	52.7	221	10.7	359	17.4	158	7.6	100	4.8
Postural instability	674	32.6	188	9.1	48	2.3	180	8.7	123	5.9	133	6.4
Freezing phenomena	127	6.1	16	0.8	2	0.1	33	1.6	39	1.9	37	1.8
Falls	55	2.7	24	1.2	12	0.6	15	0.7	3	0.1	1	0.0
Hallucinations unrelated to medication	12	0.6	1	0.0	0	0.0	4	0.2	1	0.0	6	0.3
Severe autonomic dysfunction	159	7.7	33	1.6	15	0.7	43	2.1	38	1.8	30	1.5
Dementia	223	10.8	22	1.1	16	0.8	77	3.7	52	2.5	55	2.7
First PD medication used	2052	99.2	1149	55.6	372	18.0	371	17.9	93	4.5	52	2.5
Non-IPD Parkinsonism (*N* = 378)^b,e,f^												
Tremor	241	63.8	204	54.0	17	4.5	13	3.4	5	1.3	1	0.3
Rigidity	282	74.6	196	51.9	25	6.6	45	11.9	14	3.7	2	0.5
Bradykinesia	259	68.5	173	45.8	33	8.7	38	10.1	11	2.9	4	1.1
Postural instability	166	43.9	92	24.3	26	6.9	40	10.6	8	2.1	0	0.0
Freezing phenomena	25	6.6	8	2.1	0	0.0	14	3.7	3	0.8	0	0.0
Falls	75	19.8	45	11.9	14	3.7	14	3.7	2	0.5	0	0.0
Hallucinations unrelated to medication	34	9.0	15	4.0	5	1.3	8	2.1	5	1.3	1	0.3
Severe autonomic dysfunction	83	22.0	35	9.3	7	1.9	31	8.2	10	2.6	0	0.0
Dementia	100	26.5	42	11.1	11	2.9	30	7.9	15	4.0	2	0.5
First PD medication used	300	79.4	203	53.7	41	10.8	42	11.1	8	2.1	1	0.3

^a^Missing onset dates: tremor 2/1750; rigidity 6/1977; bradykinesia 4/1932; postural instability 2/674; dementia 1/223; first PD medication use 15/2052; first antidepressants use 120/2068.

^b^Missing onset dates: tremor 1/241; first PD medication use 5/300; first antidepressant use 33/378.

^c^A total of 823 IPD cases ever took antidepressants (153 (7.4%) before and 550 (26.6%) after first PD symptoms occurred).

^d^For 1,826 (88.3%) asymmetrical onset was reported.

^e^A total of 172 non-IPD cases ever took antidepressants (50 (13.2%) before and 89 (23.5%) after first PD symptoms occurred).

^f^For 243 (64.3%) non-IPD cases asymmetrical onset was reported.

**Table 3:** Comorbidities reported in the National Hospital Register by type of Parkinsonism prior to Parkinsonism diagnosis or interview.

Group	Diagnosis	Total	Linked to National	Heart disease^a^			CVD^a^			Peripheral vascular disease^a^			Diabetes^a^			Cancer^a^			COPD^a^			Dementia^a^		
			Hospital Register	*N*	%	*P* value^b^	*N*	%	*P* value^b^	*N*	%	*P* value^b^	*N*	%	*P* value^b^	*N*	%	*P* value^b^	*N*	%	*P*-value^b^	*N*	%	*P*-value^b^
Cases	IPD	2068	2068	179	8.7	0.047	247	11.9	<0.0001	101	4.9	0.178	110	5.3	0.742	235	11.4	0.921	128	6.2	0.122	126	6.1	<0.0001
	IPD interviewed	1813	1813	152	8.4	0.102	183	10.1	0.005	88	4.9	0.205	89	4.9	0.808	199	11.0	0.783	103	5.7	0.033	76	4.2	<0.0001
	LBD	53	53	4	7.5	0.784	12	22.6	0.001	7	13.2	0.006	4	7.5	0.348	9	17.0	0.197	2	3.8	0.428	34	64.2	<0.0001
	MSA	44	44	6	13.6	0.125	3	6.8	0.232	1	2.3	1.000	1	2.3	0.723	5	11.4	1.000	4	9.1	0.566	2	4.5	0.0215
	PSP + CBD	21	21	1	4.8	1.000	5	23.8	0.018	1	4.8	0.578	3	14.3	0.092	2	9.5	1.000	2	9.5	0.667	3	14.3	0.0002
	Sec. + other types of Parkinsonism	125	125	21	16.8	<0.0001	55	44.0	<0.0001	11	8.8	0.010	13	10.4	0.011	18	14.4	0.287	14	11.2	0.126	23	18.4	<0.0001
	Essential tremor	35	35	8	22.9	0.003	10	28.6	0.0002	4	11.4	0.053	7	20.0	0.002	3	8.6	0.791	9	25.7	0.001	0	0.0	1.0000

Enrolled controls	NA	1887	1829	127	6.9		136	7.4		73	4.0		93	5.1		206	11.3		136	7.4		8	0.4	

^a^Comorbidities were defined as ever diagnosis of each type of disease in the National Hospital Register (HNR) from 1977 to 2009.

^b^Pearson's chi-square test was performed to compare the proportions of each comorbidity between Parkinsonism cases and enrolled controls. For comparisons where 25% of the cells have expected counts less than 5, Fisher's exact was performed instead.

**Table 4:** Diagnostic validity of clinical features reported in medical records within 1 year of diagnosis for 2,068 IPD and 378 non-IPD cases.

Criteria^a^	IPD (*N* = 2,068)		Non-IPD (*n* = 378)		*χ* ^2^ *P* value^b^	Sensitivity	Specificity	Positive predictive value	Negative predictive value
	*N*	%	*N*	%					
Symptoms									
Tremor	1395	67.5	221	58.5	0.0008	0.67	0.42	0.86	0.19
Rigidity	1352	65.4	221	58.5	0.0080	0.65	0.42	0.86	0.18
Bradykinesia	1311	63.4	206	54.5	0.0009	0.63	0.46	0.86	0.19
Postural instability	236	11.4	118	31.2	<0.0001	0.11	0.69	0.67	0.12
Asymmetrical onset	1761	85.2	226	59.8	<0.0001	0.85	0.40	0.89	0.33
Dementia	38	1.8	53	14.0	<0.0001	0.02	0.86	0.42	0.14
Severe autonomic dysfunction	48	2.3	42	11.1	<0.0001	0.02	0.89	0.53	0.14
Falls	36	1.7	59	15.6	<0.0001	0.02	0.84	0.38	0.14
Fast time to progression	8	0.4	27	7.1	<0.0001	0.00	0.93	0.23	0.15
Sudden symptoms	104	5.0	68	18.0	<0.0001	0.05	0.82	0.60	0.14
Hallucinations unrelated to medication	1	0.0	20	5.3	<0.0001	0.00	0.95	0.05	0.15
Freezing phenomena	18	0.9	8	2.1	0.0299	0.01	0.98	0.69	0.15
Babinski's sign	41	2.0	28	7.4	<0.0001	0.02	0.93	0.59	0.15
Supranuclear gaze palsy	7	0.3	13	3.4	<0.0001	0.00	0.97	0.35	0.15
Medications used									
Levodopa	1027	49.7	213	56.3	0.0108	0.50	0.44	0.83	0.14
Agonist ergoline	309	14.9	33	8.7	0.0009	0.15	0.91	0.90	0.16
Agonist nonergoline	664	32.1	71	18.8	<0.0001	0.32	0.81	0.90	0.18
Amantadine	27	1.3	15	4.0	0.0003	0.01	0.96	0.64	0.15
COMT inhibitor	240	11.6	35	9.3	0.1576	0.12	0.91	0.87	0.16
MAO-B	336	16.2	23	6.1	<0.0001	0.16	0.94	0.94	0.17
Multiple criteria									
Conventional criteria (at least 2 of T, R, and B^c^)	1713	82.8	261	69.0	<0.0001	0.83	0.31	0.87	0.25
All 3 cardinal features (T, R, and B)	1360	65.8	161	42.6	<0.0001	0.66	0.57	0.89	0.23
Asymmetrical onset and atypical features^d^	1836	88.8	309	81.7	<0.0001	0.89	0.18	0.86	0.23

^a^Missing onset dates: tremor (3); rigidity (6); bradykinesia (4); postural instability (2); asymmetry (4); first L-dopa treatment (44); first ergoline agonist treatment (91); first nonergoline agonist treatment (65); first amantadine treatment (11); first COMT inhibitor treatment (42); first MAO-B inhibitor treatment (344).

^b^ *χ* ^2^ test comparing the proportion of cases with iPD having the clinical features.

^c^T = tremor; R = rigidity; B = bradykinesia.

^d^Atypical features included dementia, early falls, severe symptomatic dysautonomia, fast time to progression, sudden symptoms, hallucination unrelated to medications, freezing phenomena, Babinski's sign, and supranuclear gaze palsy.

## References

[1] Gelb D. J., Oliver E., Gilman S. (1999). Diagnostic criteria for Parkinson disease. *Archives of Neurology*.

[2] Newman E. J., Breen K., Patterson J., Hadley D. M., Grosset K. A., Grosset D. G. (2009). Accuracy of Parkinson's disease diagnosis in 610 general practice patients in the West of Scotland. *Movement Disorders*.

[3] Bhidayasiri R., Reichmann H. (2013). Different diagnostic criteria for Parkinson disease: what are the pitfalls?. *Journal of Neural Transmission*.

[4] Hughes A. J., Daniel S. E., Ben-Shlomo Y., Lees A. J. (2002). The accuracy of diagnosis of parkinsonian syndromes in a specialist movement disorder service. *Brain*.

[5] Hughes A. J., Ben-Shlomo Y., Daniel S. E., Lees A. J. (1992). What features improve the accuracy of clinical diagnosis in Parkinson's disease: a clinicopathologic study. *Neurology*.

[6] Adler C. H., Beach T. G., Hentz J. G. (2014). Low clinical diagnostic accuracy of early vs advanced Parkinson disease. Clinicopathologic study. *Neurology*.

[7] Schrag A., Ben-Shlomo Y., Quinn N. (2002). How valid is the clinical diagnosis of Parkinson's disease in the community?. *Journal of Neurology, Neurosurgery and Psychiatry*.

[8] Lynge E., Sandegaard J. L., Rebolj M. (2011). The Danish national patient register. *Scandinavian Journal of Public Health*.

[9] Wermuth L., Lassen C. F., Himmerslev L., Olsen J., Ritz B. (2012). Validation of hospital register-based diagnosis of Parkinson's disease. *Danish Medical Journal*.

[10] Charlson M. E., Pompei P., Ales K. L., MacKenzie C. R. (1987). A new method of classifying prognostic comorbidity in longitudinal studies: development and validation. *Journal of Chronic Diseases*.

[11] Ritz B., Lee P., Lassen C. F., Arah O. A. (2014). Parkinson disease and smoking revisited: ease of quitting is an early sign of the disease. *Neurology*.

[12] Hughes A. J., Daniel S. E., Kilford L., Lees A. J. (1992). Accuracy of clinical diagnosis of idiopathic Parkinson's disease: a clinico-pathological study of 100 cases. *Journal of Neurology Neurosurgery and Psychiatry*.

[13] Velseboer D. C., Broeders M., Post B. (2013). Prognostic factors of motor impairment, disability, and quality of life in newly diagnosed PD. *Neurology*.

[14] Yoritaka A., Shimo Y., Takanashi M. (2013). Motor and non-motor symptoms of 1453 patients with Parkinson's disease: prevalence and risks. *Parkinsonism and Related Disorders*.

[15] Jankovic J., Rajput A. H., McDermott M. P., Perl D. P. (2000). The evolution of diagnosis in early Parkinson disease. *Archives of Neurology*.

[16] Quinn N. (1995). Parkinsonism—recognition and differential diagnosis. *British Medical Journal*.

[17] Gilman S., Wenning G. K., Low P. A. (2008). Second consensus statement on the diagnosis of multiple system atrophy. *Neurology*.

[18] Osaki Y., Ben-Shlomo Y., Lees A. J. (2004). Accuracy of clinical diagnosis of progressive supranuclear palsy. *Movement Disorders*.

[19] Stover N. P., Watts R. L. (2001). Corticobasal degeneration. *Seminars in Neurology*.

[20] Connolly B. S., Lang A. E. (2014). Pharmacological treatment of Parkinson disease: a review. *The Journal of the American Medical Association*.

[21] Christine C. W., Aminoff M. J. (2004). Clinical differentiation of Parkinsonian syndromes: prognostic and therapeutic relevance. *American Journal of Medicine*.

