# Supplementary material for: Medical Record Review to Differentiate between Idiopathic Parkinson's Disease and Parkinsonism: A Danish Record Linkage Study with 10 Years of Follow-Up
Source: Parkinsons Dis. 2015 Dec 3;2015:781479. doi: 10.1155/2015/781479 (PMC4681800; doi:10.1155/2015/781479)

Supplementary Table 1. Diagnostic value of clinical features in iPD cases and non-iPD cases based on symptoms and medications (ever/never listed in medical records)

Criteria	IPD (N=2,068)		Non-IPD (n=378)		χ² P-value[a]	Sensitivity	Specificity	Positive predictive value	Negative predictive value
	N	%	N	%					
Symptoms									
Tremor	1750	84.6	241	63.8	<0.0001	0.85	0.36	0.88	0.30
Rigidity	1977	95.6	282	74.6	<0.0001	0.96	0.25	0.88	0.51
Bradykinesia	1932	93.4	259	68.5	<0.0001	0.93	0.31	0.88	0.47
Postural instability	674	32.6	166	43.9	<0.0001	0.33	0.56	0.80	0.13
Freezing phenomena	127	6.1	25	6.6	0.7264	0.06	0.93	0.84	0.15
Falls	55	2.7	75	19.8	<0.0001	0.03	0.80	0.42	0.13
Hallucinations unrelated to medication	12	0.6	34	9.0	<0.0001	0.01	0.91	0.26	0.14
Severe autonomic dysfunction	159	7.7	83	22.0	<0.0001	0.08	0.78	0.66	0.13
Dementia	223	10.8	100	26.5	<0.0001	0.11	0.74	0.69	0.13
First PD medication used	2052	99.2	300	79.4	<0.0001	0.99	0.21	0.87	0.83
Medication use									
Levodopa	1806	87.3	290	76.7	<0.0001	0.87	0.23	0.86	0.25
Agonist ergolin	752	36.4	56	14.8	<0.0001	0.36	0.85	0.93	0.20
Agonist non ergolin	1551	75.0	120	31.7	<0.0001	0.75	0.68	0.93	0.33
Amantadin	249	12.0	47	12.4	0.8293	0.12	0.88	0.84	0.15
COMT inhibitor	1096	53.0	91	24.1	<0.0001	0.53	0.76	0.92	0.23
MAO-B	1053	50.9	79	20.9	<0.0001	0.51	0.79	0.93	0.23
Multiple criteria									
Conventional criteria (at least 2 of T, R, B[b])	2011	97.2	279	73.8	<0.0001	0.97	0.26	0.88	0.63
All 3 cardinal features (T, R, B)	1592	77.0	171	45.2	<0.0001	0.77	0.55	0.90	0.30
Asymmetrical onset and atypical features[c]	1898	91.8	321	84.9	<0.0001	0.92	0.15	0.86	0.25

[a] χ² test comparing the proportion of cases with iPD having the clinical features.
[b] T=Tremor; R=Rigidity; B= Bradykinesia.
[c] Atypical features included dementia, early falls, severe symptomatic dysautonomia, fast time to progression, sudden symptoms, hallucination unrelated to medications, freezing phenomena, Babinski's sign and supranuclear gaze palsy.

Supplementary Table 2. Medical treatment registered in charts at any time

Diagnosis	Total	Levodopa		Agonist ergolin		Agonist non ergolin		COMT inhibitor		MAO-B	
		N	%	N	%	N	%	N	%	N	%
IPD	2068	1806	87.3	752	36.4	1551	75.0	1096	53.0	1053	50.9
IPD-interviewed	1813	1560	86.0	624	34.4	1391	76.7	932	51.4	918	50.6
LBD	53	50	94.3	2	3.8	24	45.3	15	28.3	13	24.5
MSA	44	44	100.0	14	31.8	31	70.5	23	52.3	22	50.0
PSP + CBD	21	21	100.0	5	23.8	6	28.6	6	28.6	3	14.3
Sec + other Parkinsonism	125	112	89.6	25	20.0	45	36.0	40	32.0	29	23.2
Essential tremor	35	20	57.1	7	20.0	5	14.3	2	5.7	5	14.3

Supplementary Figure 1. ROC curves for diagnosing IPD based on symptoms and PD medications (listed in medical records within 1 year after diagnosis)

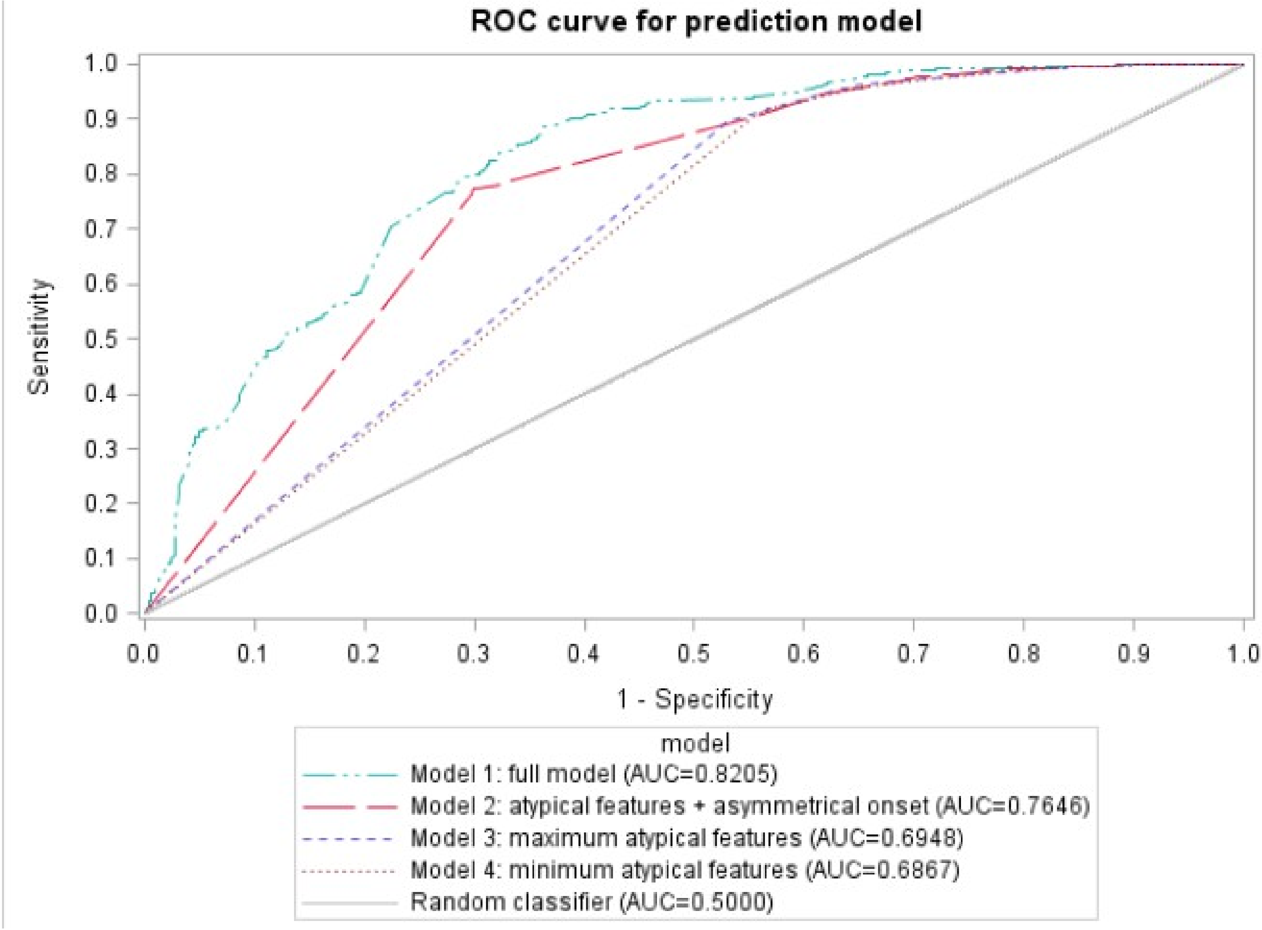

Supplement: Supplementary file 1 — Fig. 1. shows the ROC curves for diagnosing IPD after 1 year based on symptoms and PD medications. Table 1. shows the diagnostic value of clinical features in IPD cases and non-IPD cases based on symptoms and medications. Table 2. shows the medical treatment registered in medical records at any time. [file 781479.f1.pdf]
